# Robust plant disease segmentation in complex field environments: an in-depth analysis and validation with STAR-Net

**DOI:** 10.3389/fpls.2025.1706072

**Published:** 2026-01-28

**Authors:** Yulong Fan, Minghao Yu, Lele Shen, Jie Ma, Zhisheng Zeng, Hui Wang

**Affiliations:** College of Computer Science and Technology, Zhejiang Normal University, Jinhua, China

**Keywords:** plant disease segmentation, deep learning, Swin Transformer, attention mechanism, loss function, precision agriculture

## Abstract

**Introduction:**

Plant disease segmentation in real-world agricultural environments poses significant technical challenges, including complex backgrounds, diverse lesion morphologies, and extreme class imbalance.

**Methods:**

In this paper, we propose an integrated solution, STAR-Net, which combines a novel network architecture with a dynamic training strategy. The architecture features an innovative Heterogeneous Branch Attention Aggregation (HBAA) module to robustly represent multi-scale and multi-morphology features. The training strategy employs a Dynamic Phase-Weighted Loss (DPW-Loss) to navigate the complexities of imbalanced data.

**Results:**

Our method achieves a state-of-the-art average mIoU of 93.36% on the NLB dataset. This result demonstrates its superior ability to precisely segment diseases with specific elongated morphologies. Furthermore, the model obtains a competitive average mIoU of 41.13% on the highly challenging PlantSeg dataset. This result validates its robustness in complex 'in-the-wild' scenarios.

**Discussion:**

Our work presents a powerful, well validated, and synergistic solution for plant disease segmentation. It also paves the way for practical applications in precision agriculture.

## 1 Introduction

Agriculture is a cornerstone of global food security, but plant diseases threaten its sustainability. According to the Food and Agriculture Organization (FAO), these diseases can cause annual crop losses of up to 40% (Fao, 2019). To mitigate these losses, precision agriculture aims to replace broad-scale treatments with targeted interventions. This paradigm relies on a synergistic workflow. Front-end technologies, like the Internet of Things (IoT) and drone imaging, acquire data. Subsequently, advanced computer vision models provide the intelligent analysis needed for decision-making (Khan et al., 2022a; Bashir et al., 2023; Khan et al., 2022b; Luo et al., 2022). Accurate and automated segmentation of plant diseases is a critical component of this workflow. It enables precise pesticide application. This practice reduces environmental pollution and enhances crop yield and quality (Kamilaris and Prenafeta-Boldú, 2018; Barbedo, 2018). Driven by advances in deep learning, vision-based analysis now offers powerful support for the early warning and precise control of plant diseases (Kamilaris and Prenafeta-Boldú, 2018; Mohanty et al., 2016; Parashar et al., 2024; Van Klompenburg et al., 2020; Vishnoi et al., 2022; Chen et al., 2025).

However, deploying deep learning models for plant disease segmentation in real-world agricultural environments—often termed “in-the-wild”—presents several formidable challenges. First, field images inherently possess high complexity. Unlike in controlled settings, they contain varied backgrounds (soil, weeds), dynamic lighting (shadows, reflections), and occlusions. Plants also exhibit morphological changes across growth stages, with disease symptoms that can be subtle and varied (Wei et al., 2024; Barbedo, 2018). Second, the visual characteristics of diseases are complex. They exhibit high intra-class variation, where one disease appears differently, and high inter-class similarity, where different diseases appear similar. Third, agricultural data suffer from extreme class imbalance. Healthy plant regions typically dominate diseased pixels, and the prevalence of different diseases varies enormously. For instance, in a large-scale dataset featuring 115 diseases, the pixel count of the rarest classes can be thousands of times smaller than that of common ones (Wei et al., 2024). This imbalance biases models toward majority classes, causing poor performance on rare diseases or small lesions. Finally, precision agriculture demands pixel-level accuracy to quantify disease severity. This requires models to both localize lesions and precisely delineate their boundaries. This task is made even more difficult by diverse lesion morphologies, like the elongated patterns of Northern Leaf Blight in maize.

To address these challenges, researchers have adapted various deep learning models for agricultural scenes, though with notable limitations. Classic architectures like U-Net (Ronneberger et al., 2015) are effective in some contexts. However, they often struggle with complex field images because of their limited receptive fields. Models designed for multi-scale feature capture, such as PSPNet (Zhao et al., 2017) and DeepLabV3+ (Chen et al., 2018) with its Atrous Spatial Pyramid Pooling (ASPP) module, offer improvements. However, these models often require task-specific modifications, like adding extra attention modules for irregular lesions. This approach results in “patchwork” solutions (Peng et al., 2023). The task is so difficult that some researchers adopt cumbersome two-stage approaches: segmenting leaves first, then lesions. This highlights the challenge for a single model to handle both complex backgrounds and fine-grained targets (Zhu et al., 2023; Xu et al., 2025). Even modern Transformer-based architectures like SegFormer (Xie et al., 2021) can struggle to balance global context with fine detail recovery (Guan et al., 2025).

Furthermore, a recent and dominant trend is the rise of large-scale, domain-specific foundation models for agriculture, such as those pre-trained on massive datasets for various agri-vision tasks (Mehdipour et al., 2025; Nahian, 2025). These models, often based on Vision Transformer (ViT) architectures, demonstrate powerful generalization capabilities by learning rich, transferable representations (Li et al., 2025).

However, this paradigm introduces its own set of challenges. First, these large foundation models often carry significant computational overhead and require vast amounts of pre-training data, making them difficult to train and deploy in resource-constrained precision agriculture scenarios, such as on drones or edge devices (Mehdipour et al., 2025). Second, while effective for general tasks like whole-plant classification or land-cover mapping, they may still require significant fine-tuning or specialized modules to handle the specific, fine-grained, and highly imbalanced nature of *plant disease lesion segmentation* (Quoc et al., 2025; Prashanth et al., 2024).

These persistent challenges—ranging from the limitations of classic CNNs to the high cost and generalization gaps of recent foundation models—suggest a fundamental need for a targeted, end to-end model. Such a model must better balance accuracy, efficiency, and detail fidelity in complex agricultural scenes (Yang et al., 2022; Zhou et al., 2024, 2025).

Meanwhile, the design of the loss function is equally critical for optimizing deep learning models for plant disease segmentation. Standard Cross-Entropy (CE) loss is a common choice but struggles with class imbalance. Because healthy regions dominate, the model often neglects minority lesion classes (Lin et al., 2017). Dice loss and its generalized form (GDL) directly optimize regional overlap (Sudre et al., 2017). However, their gradients can become unstable when dealing with numerous small lesions. Focal Loss (FL) addresses imbalance by down-weighting easy samples (Lin et al., 2017). However, its static nature may not adapt to the model’s evolving learning state or the diversity of lesion sizes.

In summary, a clear research gap exists for a solution that synergistically combines architectural innovation with an intelligent training strategy. We aim to answer two core questions. First, can we design a network that efficiently aggregates multi-scale, heterogeneous features to handle complex visual patterns? Second, can we construct a loss function that dynamically adapts its optimization target to overcome class imbalance? To holistically tackle these challenges, this paper proposes STAR-Net, an integrated solution. It combines a novel network architecture featuring a Heterogeneous Branch Attention Aggregation (HBAA) module with an intelligent training strategy, the Dynamic Phase-Weighted Loss (DPW-Loss).

The main contributions of this paper are as follows:

1. A novel segmentation architecture, STAR-Net, which uses a Swin Transformer backbone and an innovative Heterogeneous Branch Attention Aggregation (HBAA) module to effectively capture multi-scale, multi-morphology disease features in complex backgrounds.
2. A dynamic training strategy, DPW-Loss, which adaptively adjusts the weights of multiple loss functions based on training phases and validation feedback, effectively addressing extreme class imbalance and guiding the model toward robust convergence.
3. Comprehensive experimental validation of our method against several mainstream models on three diverse datasets, including the highly challenging “in-the-wild” PlantSeg dataset. The results demonstrate the superior performance and robustness of our integrated approach, particularly in handling specific lesion morphologies and complex, imbalanced scenarios.

## 2 Methods and datasets

This section first describes the datasets used in our study, including their sources, characteristics, and preprocessing methods. We then present the STAR-Net deep learning model architecture, which was designed to address the specific challenges of plant disease segmentation. This includes the design philosophy and implementation of its core components. Finally, we introduce the DPW-Loss, a dynamic phase-weighted loss function used to optimize the model training process.

### 2.1 Datasets

Our study uses three plant disease datasets: a self-developed Apple Disease Leaf Dataset (ADLD) and two public datasets (PlantSeg and NLB). These datasets vary in crop type, disease complexity, image acquisition environment, and annotation quality. These datasets provide a solid foundation for evaluating our model’s performance, robustness, and generalization.

#### 2.1.1 Apple Disease Leaf Dataset

The Apple Leaf Disease Dataset (ADLD) is a self-developed dataset focusing on apple leaf diseases. It covers five common diseases: Apple scab, Black rot, Cedar apple rust, Alternaria blotch, and Rust. To build this dataset, we collected 868 original disease images from open-source communities like Kaggle and other web resources. We manually performed pixel-level annotation using the LabelMe software. All images underwent a unified data augmentation and preprocessing pipeline, resulting in a final dataset of 11,284 images. The class distribution and the split between training and validation sets are shown in **Table 1**.

**Table 1:** Image distribution of each class in the ADLD dataset.

Disease category	Original	Augmented train	Augmented val	Augmented total
Alternaria Blotch	140	1626	193	1819
Apple Scab	155	1810	205	2015
Black Rot	162	1915	191	2106
Cedar Apple Rust	97	1150	111	1261
Rust	314	3655	428	4083
Total	868	10156	1128	11284

#### 2.1.2 PlantSeg dataset

The PlantSeg dataset (Wei et al., 2024) is a large-scale plant disease segmentation dataset captured in real-world, “in-the-wild” field environments. Its key feature is that all images were collected in authentic field conditions. These images include complex variations in lighting (e.g., strong light, shadows), backgrounds (e.g., soil, weeds), and extensive occlusions. According to its authors, the dataset covers 115 diseases across 34 crop species and contains over 11,400 images with fine-grained pixel-level annotations. The PlantSeg dataset is complex and large-scale. It provides a valuable platform for testing a model’s robustness, generalization, and ability to handle real-world agricultural challenges.

#### 2.1.3 Northern Leaf Blight dataset

The NLB dataset (Prashanth et al., 2023) is a public dataset focused on Northern Leaf Blight in maize. It contains 1,000 images of corn leaves taken under field conditions. The dataset also provides high-quality lesion segmentation masks that were manually refined by domain experts. This dataset is designed to evaluate a model’s ability to identify specific diseases. It is particularly useful for diseases with distinct morphologies, such as long, spindle-shaped lesions. It represents a real-world segmentation task for a major food crop.

For model training and evaluation, all datasets were split into training (80%), validation (10%), and test (10%) sets. The detailed class distribution for our self-developed ADLD dataset is shown in **Table 2**.

**Table 2:** Summary of datasets used in the experiment.

Dataset	Source/citation	Classes	Total images	Collection environment	Annotation quality	Core challenges
ADLD	Constructed in this study (see **Table 1**)	6	11,284	Diverse public sources	Pixel-level	Evaluating the model’s basic segmentation performance in specific scenarios.
PlantSeg	Wei et al (Wei et al., 2024)	115	11,400+	Real field conditions	Pixel-level	Complex lighting/backgrounds, multi-class imbalance, and fine-grained lesions.
NLB	Prashanth et al (Prashanth et al., 2023)	2	1000	Real field conditions	Manually refined by experts	Identifying and quantifying diseases with specific morphologies (e.g. elongated spindle shape).

Before being fed into the model, all images underwent a unified preprocessing pipeline. This process first resizes all input images and their annotations to a fixed size of 512 × 512 pixels. To enhance the model’s generalization ability and prevent overfitting, we then applied a data augmentation strategy. This strategy was randomly applied to input images during each training iteration. The specific augmentations were: random multi-scale scaling (range 0.5 to 2.0), random 512×512 cropping, and random horizontal flipping (probability 0.5). We also applied photometric distortion to simulate different lighting conditions. Finally, the pixel values of the augmented images were normalized. These steps are crucial for ensuring data consistency, expanding the diversity of training samples, and improving the model’s final performance.

### 2.2 STAR-Net model architecture

**Figure 1:**
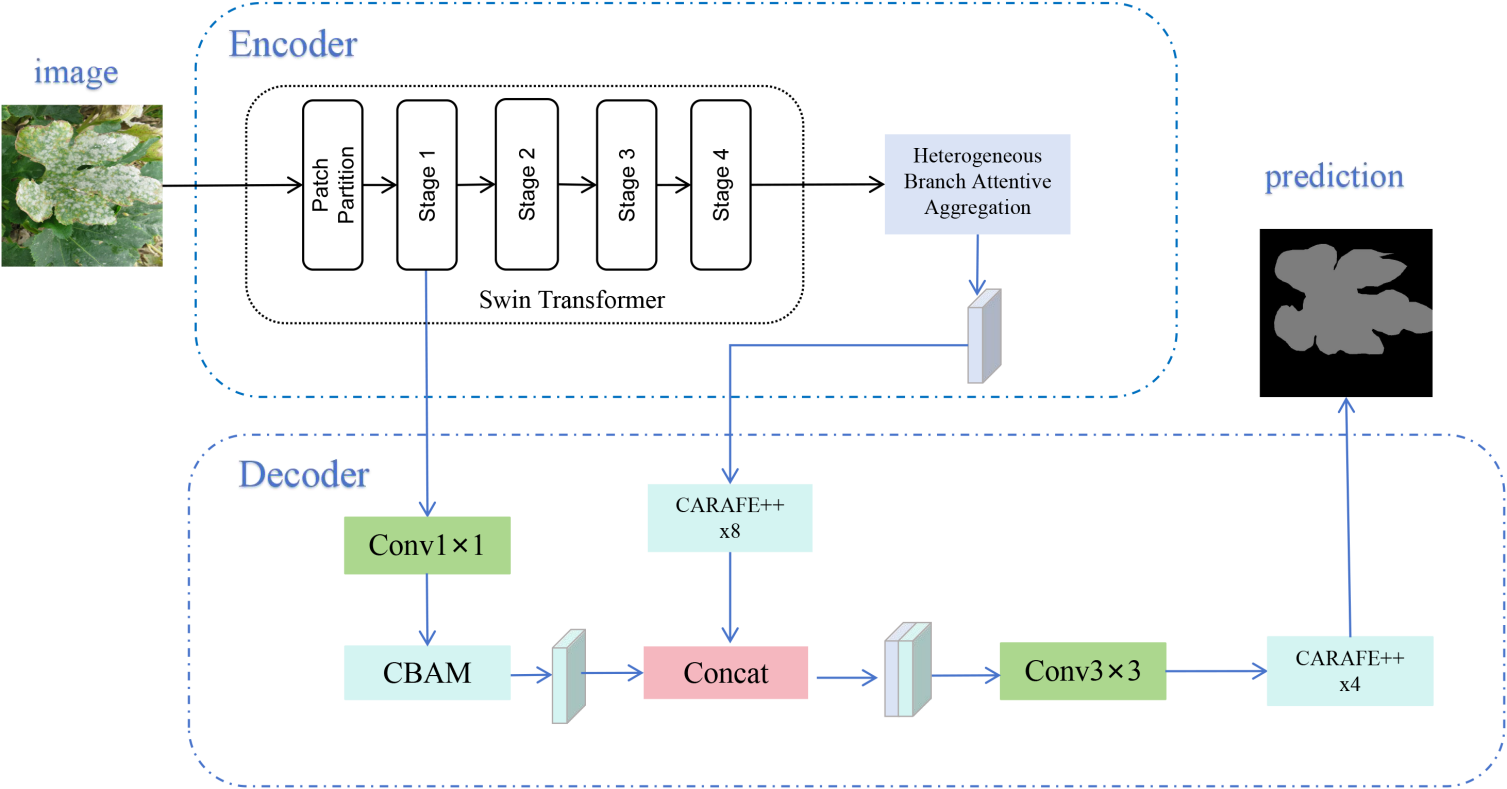
The overall architecture of STAR-Net. The model consists of a Swin Transformer-based encoder and a fusion decoder. The encoder enhances deep features through the HBAA module, while the decoder uses CARAFE++ for upsampling to fuse shallow details with deep semantic information.

We designed STAR-Net to address the inherent challenges in plant disease images, such as complex backgrounds, variable lighting, and diverse lesion shapes and sizes. It is a deep learning model with a classic Encoder-Decoder structure. The model robustly extracts and integrates multi-scale features from complex agricultural images. Its goal is to achieve precise pixel-level segmentation of diseased regions. Its overall architecture is shown in **Figure 1**.

#### 2.2.1 Encoder design: feature extraction and enhancement

The core task of the STAR-Net encoder is to efficiently capture key visual information from the input image and generate discriminative feature representations. It primarily consists of a Swin Transformer backbone and our proposed Heterogeneous Branch Attention Aggregation (HBAA) module.

##### 2.2.1.1 Swin Transformer backbone

We chose the Swin Transformer (Liu et al., 2021) as the feature extraction backbone for STAR-Net. This choice was based on three key properties. First, it has a powerful multi-scale feature representation capability. Its hierarchical design builds feature maps at different scales. This is crucial for accurately segmenting plant diseases of various sizes and developmental stages. Second, it possesses efficient global context modeling. Its core shifted window self-attention mechanism effectively models an image’s global context and limits computational complexity. This is particularly important in complex, uncontrolled “in-the-wild” environments, as it helps the model better distinguish disease features from background noise. Finally, the Swin Transformer is suitable for high-resolution image processing. Its computational complexity is linear for high-resolution images. This makes it well-suited for high-definition agricultural images where detail preservation is key.

In STAR-Net, we use outputs from two specific stages of the Swin Transformer. Features from Stage 1, at 1/4 of the original input resolution, maximally preserve low-level spatial details and high-frequency edge information. This is vital for the decoder to accurately delineate lesion boundaries. Features from Stage 4, at 1/32 resolution, contain richer and more robust deep semantic information. This helps the model understand “what a diseased region is” on a macroscopic level and serves as the input for the HBAA feature enhancement module.

##### 2.2.1.2 Heterogeneous Branch Attention Aggregation module

Design Motivation and Objective: Deep features from Swin Transformer’s Stage 4 are rich in semantic information. However, they still face challenges related to scale diversity and morphological variety in complex “in-the-wild” images. While general-purpose multi-scale modules like ASPP are powerful, they may not be optimal for the specific and often anisotropic morphologies of plant diseases. Our design philosophy for the HBAA module is rooted in creating a specialized, heterogeneous feature aggregator tailored to these challenges.

**Figure 2:**
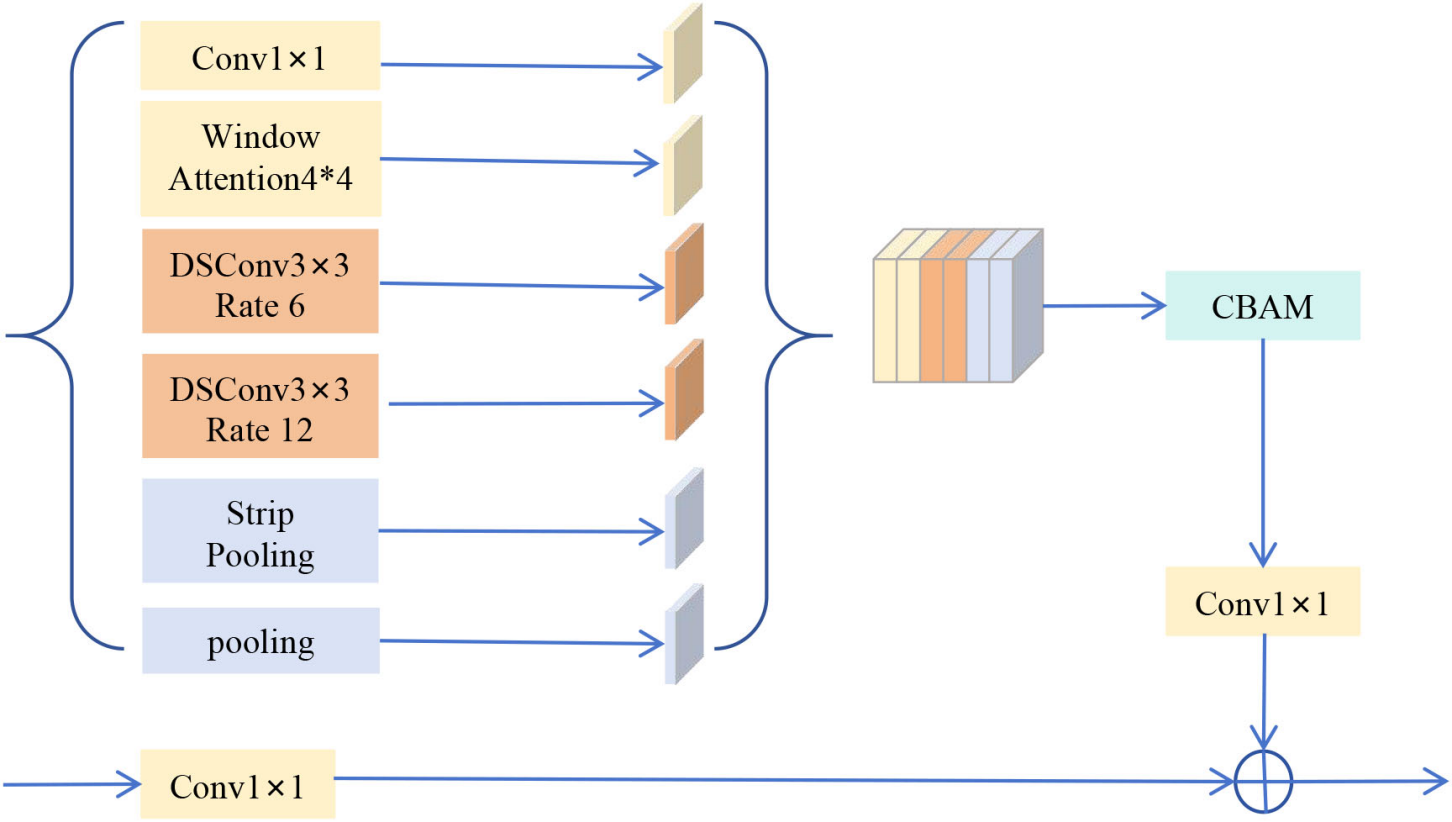
Architecture of the Heterogeneous Branch Attention Aggregation (HBAA) module. To efficiently capture diverse disease features, this module integrates several key branches in parallel: Window Attention for capturing local context, multi-dilation rate convolution for extracting multiscale information, and Strip Pooling specifically for stripe-like lesions. The features from each branch are concatenated, fused using a CBAM module, and combined with a residual connection to enhance the expression of key features.

Its core objective is to generate a more sensitive and robust feature representation. It achieves this by integrating different feature extraction operations in parallel, creating a synergy that general-purpose methods lack. For instance, the Strip Pooling branch captures the structural integrity of elongated lesions like Northern Leaf Blight. Meanwhile, the Window Attention branch focuses on their fine-grained local textures. The HBAA module processes distinct morphological and textural features in parallel and adaptively fuses them using CBAM. This process generates a more discriminative feature representation for agricultural scenes than a monolithic, general-purpose fusion module could. The detailed structure is shown in **Figure 2**.

**Figure 3:**
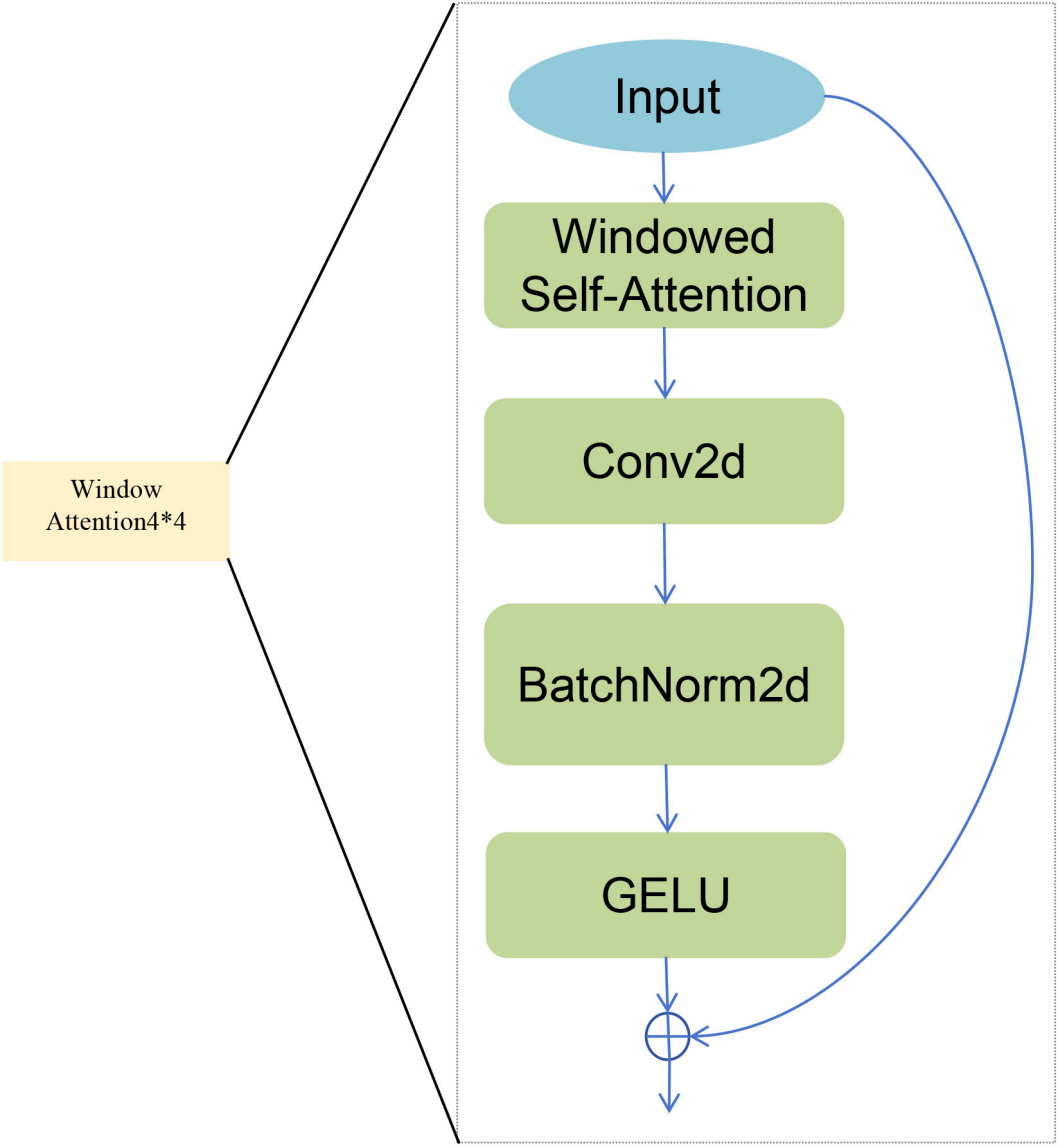
Architecture of the Window Attention branch within the HBAA module.

The HBAA module takes the deep feature map from Swin Transformer Stage 4 as input. It contains a parallel feature extraction unit designed to comprehensively capture complex disease features from multiple dimensions. This unit integrates six complementary branches. They are responsible for basic transformation, fine-grained local modeling, multi-scale context, specific morphologies, and a global summary. This combination ensures the network can perceive both the subtle textures of lesions and their relationship with the surrounding environment. The unit consists of the following six branches:

- 1x1 convolution: Provides a basic linear transformation of features.
- Window attention: Finely models complex dependencies between features within a 4x4 local window. This is particularly effective for capturing subtle textural consistency within lesions. Its structure is detailed in **Figure 3**.
- Two depthwise separable convolutions with different atrous rates (rate=6, rate=12) (Howard 203 et al., 2017; Yu and Koltun, 2015), Capture medium and large-range contextual information in a lightweight manner to identify lesions of different sizes.
- Strip Pooling (Hou et al., 2020): To specifically address the challenge of segmenting diseases with elongated morphologies, as highlighted in the introduction, we incorporate Strip Pooling. This branch is motivated by the need to model diseases like Northern Leaf Blight accurately. It aggregates features along horizontal and vertical extents to preserve the structural integrity of such lesions.
- Image pooling: This captures a global contextual summary of the feature map via global average pooling. It enhances the model’s robustness against complex backgrounds.

Feature maps from the six parallel branches are concatenated along the channel dimension to form a more comprehensive composite feature representation. The concatenated features are then fed into a Convolutional Block Attention Module (CBAM) (Woo et al., 2018) for adaptive filtering and weighting. This aims to enhance significant disease-related features while suppressing irrelevant background noise. For smooth information flow and training stability, the CBAM-processed features are passed through a final 1x1 convolutional layer. This performs fusion and channel adjustment. A residual connection is then made with the module’s original input. The HBAA module outputs a multi-enhanced and refined deep feature map, maintaining the same spatial resolution as the input.

#### 2.2.2 Decoder design: feature fusion and upsampling

The decoder’s core task is twofold: to fuse the multi-level features from the encoder and to progressively restore the feature map’s spatial resolution. This process generates a high-precision segmentation result. The design focuses on how to maximally utilize shallow features to recover fine lesion boundaries while preserving deep semantic guidance.

**Figure 4:**
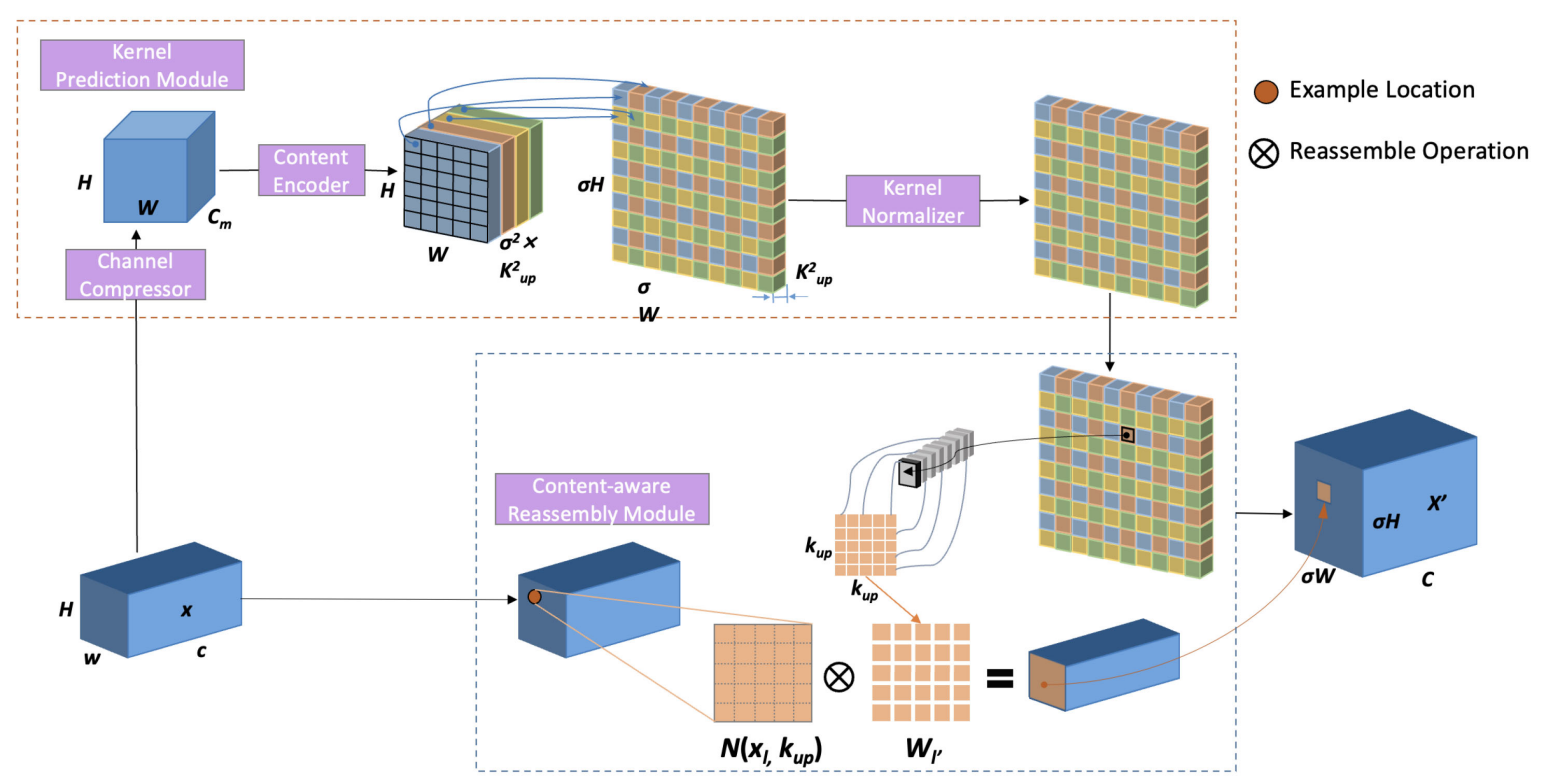
Detailed schematic of the CARAFE module architecture. This figure is reprinted from Sun et al. (2024), originally proposed by Wang et al. (2019). In the schematic, H and W denote the height and width of the input feature map, respectively, while C represents the number of input channels. C~m~ indicates the compressed channels utilized in the kernel prediction module. σ refers to the upsampling scale factor, and k~up~ × k~up~ denotes the size of the reassembly kernel.

The process begins with two parallel feature paths. In the shallow feature path, the detail-rich features from Swin Transformer Stage 1 first pass through a 1x1 convolutional layer for channel adjustment. They are then fed into a CBAM module. This enhances the most valuable shallow features for boundary identification and subtle lesion detection using an attention mechanism. Simultaneously, in the deep feature path, features from the HBAA module are upsampled by a factor of 8. We use the efficient CARAFE++ operation for this task (Wang et al., 2019). We chose CARAFE++ for its “content-aware” property. This feature significantly reduces information loss and visual artifacts when restoring complex lesion boundaries (as shown in **Figure 4**).

Next, the features from both paths are fused. The upsampled deep features and the enhanced shallow features are concatenated along the channel dimension to preserve all information. The resulting feature map then passes through a 3x3 convolutional layer for deep fusion and smoothing. This promotes effective interaction between shallow and deep information. Finally, the fused and refined features (now at 1/4 resolution) are upsampled again by a factor of 4 using CARAFE++, restoring them to the original image size. A Softmax or Sigmoid function then generates the final segmentation result. With this design, the STAR-Net decoder can better combine the global understanding of deep semantics with the localizing ability of shallow details. This should produce segmentation masks with clearer boundaries, smoother contours, and more complete internal regions.

### 2.3 DPW-Loss: a dynamic phase-weighted loss function for robust training

A powerful and sophisticated architecture like STAR-Net can only realize its full potential when guided by an equally intelligent optimization strategy. We propose the Dynamic Phase-Weighted Loss (DPW-Loss) to unlock our network’s capabilities. This is especially important for addressing the severe class imbalance and phased learning difficulties inherent in agricultural datasets.

The core idea of DPW-Loss is to mimic the phased learning of a human expert. It uses a dynamic weight adjustment mechanism based on training phase awareness and model learning state feedback.

This process is analogous to a plant pathologist’s skill development. It involves an initial phase of broad learning, a mid-phase for tackling difficult cases, and a final phase of refinement for precision. DPW-Loss dynamically adjusts the contribution of each base loss function over time. This allows each function to dominate when it is most effective, thereby achieving synergistic optimization.

DPW-Loss simulates a phased learning process using a dynamic weight adjustment mechanism. It integrates three common loss functions from semantic segmentation. First, Cross-Entropy Loss (*L_CE_*) provides a stable foundation. It offers smooth gradients during early training to help the model learn basic visual features. Second, Focal Loss (*L_Focal_*) (Lin et al., 2017) addresses class imbalance by down-weighting easy samples to force the model to focus on hard-to-classify ones. Finally, Generalized Dice Loss (*L_GDL_*) (Sudre et al., 2017) directly optimizes regional overlap, which is a more direct target for segmentation tasks.

The novelty of DPW-Loss lies in its dual-trigger, semi-automated transition logic, which is not a purely fixed schedule. The mechanism incorporates two conditions to initiate a phase transition:

1. Primary Trigger (Feedback-Driven): The main criterion is the convergence of key metrics on the validation set. For instance, the transition from Phase 1 to 2 continuously monitors the moving average of the *L_CE_*. The transition is automatically initiated once the rate of change falls below a predefined threshold. This indicates that the model’s learning has stabilized for that phase.
2. Failsafe Trigger (Pre-scheduled): The fixed iteration points (e.g., 80k and 240k iterations) serve as a ‘failsafe’ or ‘latest possible’ transition point. Their purpose is to ensure the training process does not become stuck. This guarantees overall stability if the feedback-driven trigger is not activated. In many of our experiments, the feedback-driven trigger activates the transition earlier than these failsafe iteration counts.

This ‘convergence-as-primary-trigger, fixed-iteration-as-failsafe’ mechanism accurately reflects our method’s semi-automated and feedback-driven nature.

In the first phase (foundational learning), the goal is for the model to build a basic, broad visual understanding of all classes and to ensure training stability. Here, *L_CE_* is assigned a high weight *α*(*t*), while the weights for *L_Focal_* and *L_GDL_* are low. The end of this phase is primarily determined by the convergence of the moving average of *L_CE_* on the validation set. Once the model has basic classification ability, it enters the second phase (addressing difficult cases and imbalance). The focus shifts to handling extreme class imbalance and distinguishing visually similar diseases. We smoothly decrease the weight of *α*(*t*) while significantly increasing the weight of *L_Focal_*, *γ*(*t*), making it dominant. This forces the model to concentrate on difficult samples. Finally, when the model can distinguish between disease classes well, it enters the third phase (boundary refinement and overlap maximization). This transition is based on monitoring the convergence of *L_Focal_* and the mean Intersection over Union (mIoU) on the validation set. Only when the mIoU exceeds a preset threshold *T_mIoU_*___*_target_* do we significantly increase the weight of *L_GDL_*, *β*(*t*). This makes it the dominant factor in the late training stages to refine segmentation boundaries and maximize overlap with the ground truth.

**Figure 5:**
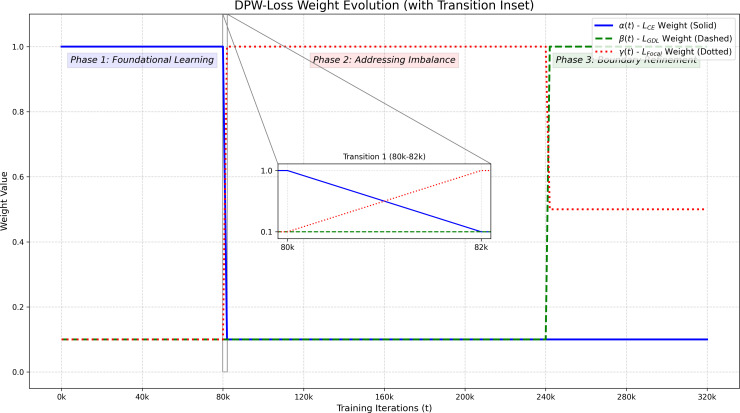
The dynamic evolution of DPW-Loss weights *α*(*t*), *β*(*t*), and *γ*(*t*) over 320k training iterations. The plot shows the three main phases (Phase 1: *L_CE_* dominant; Phase 2: *L_Focal_* dominant; Phase 3: *L_GDL_* dominant). The inset plots (magnified views) clearly show the smooth 2,000-iteration linear interpolation during phase transitions, which avoids sudden shocks to the training process.

The weights *α*(*t*)*, β*(*t*)*,γ*(*t*) are therefore best described as piecewise functions defined by the training iteration *t*. Their dynamic evolution, including the smooth, linear interpolation during phase transitions, is visualized in **Figure 5**.

#### 2.3.1 Implementation details

The implementation of our dual-trigger mechanism over 320k training iterations is as follows:

- Phase 1 (Foundational Learning, 0 - ~80k iterations): The training starts by prioritizing stable learning. The loss weights are initialized to *α* = 1.0*, β* = 0.1, and *γ* = 0.1, making the Cross-Entropy loss (*L_CE_*) the dominant factor.
- Transition to Phase 2: The transition is primarily triggered when the validation *L_CE_*loss converges (specifically, when its rate of change drops below 0.005 over a moving window of the last 10,000 iterations). As a failsafe, this transition is initiated no later than the 80,000 iteration mark. Upon triggering, the weights are smoothly adjusted via linear interpolation over the next 2,000 iterations.
- Phase 2 (Addressing Imbalance, ~80k - ~240k iterations): The focus shifts to tackling hard samples and class imbalance. The weights are transitioned to *α* = 0.1*, β* = 0.1, and *γ* = 1.0, making the Focal Loss (*L_Focal_*) dominant.
- Transition to Phase 3: This transition is primarily triggered based on the convergence of *L_Focal_* and the mIoU on the validation set (specifically, when the mIoU exceeds our preset threshold of *T_mIoU_*___*_target_* = 0.39 (39.0%)). As a failsafe, it is initiated no later than the 240,000 iteration mark.
- Phase 3 (Boundary Refinement, ~240k - 320k iterations): In the final stage, the priority is to refine segmentation boundaries. The weights are adjusted toward *α* = 0.1*, β* = 1.0, and *γ* = 0.5, making the Generalized Dice Loss (*L_GDL_*) the primary optimization target.

#### 2.3.2 Mathematical formulation

The total loss function can be expressed as Equation 1:

(1) $$L_{DPW}(t)=\alpha (t)\cdot L_{CE}+\beta (t)\cdot L_{GDL}+\gamma (t)\cdot L_{Focal}$$

where t represents the training progress. The coefficients *α*(*t*)*, β*(*t*)*,γ*(*t*) change dynamically with the training progress t and the phase-transition logic described above. To ensure training stability, the weight adjustments during phase transitions are not instantaneous. Instead, they are smoothed over a predefined transition period using linear interpolation. This mechanism ensures the continuity of the optimization objective. It also effectively avoids training oscillations from sudden weight changes.

#### 2.3.3 Core innovation of DPW-Loss

In summary, the core innovation of DPW-Loss lies in its phase-aware synergistic mechanism. This mechanism explicitly divides the training process into stages with different challenges. A dynamic weight adjustment strategy, based on validation feedback, allows different loss functions to perform their roles at optimal times. *L_CE_* is responsible for stable initial learning, while *L_Focal_* takes over in the mid-phase to tackle class imbalance and difficult samples. This dynamic guidance differs from traditional fixed-epoch scheduling. It allows DPW-Loss to more intelligently guide advanced networks like STAR-Net through the challenges of plant disease segmentation.

## 3 Experimental evaluation

We conducted detailed experiments to comprehensively evaluate the performance of our STAR-Net model and DPW-Loss function. This section details the evaluation metrics, implementation specifics, and baseline methods for comparison. It also presents and analyzes the experimental results.

### 3.1 Evaluation metrics

We primarily use two widely adopted metrics in semantic segmentation to quantitatively evaluate our model and compare it fairly with baseline methods.

The first is Mean Intersection over Union (mIoU), which is one of the most central and representative metrics for this task. It first calculates the ratio of intersection to union between the predicted and ground truth regions for each class (the IoU for that class). It then computes the arithmetic mean of the IoU values across all classes. The formula is:


$$IoU_{c}=\frac{TP_{c}}{TP_{c}+FP_{c}+FN_{c}},$$

$$mIoU=\frac{1}{N_{c}}\sum_{c=1}^{N_{c}}IoU_{c}$$

where *TP_c_* (True Positives) is the number of pixels correctly predicted for class c, *FP_c_* (False Positives) is the number of pixels from other classes incorrectly predicted as class c, and *FN_c_* (False Negatives) is the number of pixels from class c incorrectly predicted as other classes. *N_c_* is the total number of classes, including the background. mIoU effectively reflects the model’s overall segmentation accuracy and its alignment with the ground truth. This metric is especially critical for tasks that require precise boundary delineation. In cases of class imbalance, mIoU provides a more objective evaluation than pixel accuracy.

The second metric is Mean Pixel Accuracy (mAcc or MPA). mAcc first calculates the proportion of correctly classified pixels within each class relative to the total number of actual pixels in that class (i.e., the pixel accuracy or recall for each class). It then takes the arithmetic mean of these accuracies across all classes. The formula is:


$$PixelAccuracy_{c}=\frac{TP_{c}}{TP_{c}+FN_{c}},$$

$$mAcc=\frac{1}{N_{c}}\sum_{c=1}^{N_{c}}PixelAccuracy_{c}$$

While mAcc provides pixel-level classification accuracy, it can be misleading. In cases of significant class imbalance, the high accuracy of the majority class can dominate the metric, leading to an overly optimistic assessment of minority class performance. Therefore, mIoU is generally considered a more central metric for segmentation tasks, but mAcc serves as an important supplementary reference. In reporting our results, we will primarily use mIoU and mAcc for performance comparison and discussion.

### 3.2 Experimental setup

All experiments were conducted on a server equipped with an Intel Core i9-13900K CPU and two NVIDIA GeForce RTX 4090 GPUs, running the Windows 11 operating system. The experiments were based on the PyTorch 1.12.0 deep learning framework and accelerated with CUDA 10.2 and cuDNN 8.0.1.

We used the AdamW optimizer (Loshchilov and Hutter, 2017) to train the models. The initial learning rate was set to $6\times 10^{-5}$, with betas parameters of (0.9, 0.999) and a weight decay of 0.01. We employed a polynomial learning rate decay policy (power=0.9). Over a total of 320,000 training iterations, the learning rate gradually decreased from its initial value to a target minimum of $\eta_{min}=1\times 10^{-6}$. The batch size used for training was 4.

To accelerate convergence and enhance performance, the Swin Transformer backbone of STAR-Net and all other ResNet or MiT-based baseline models were initialized with weights pre-trained on ImageNet-1K. The only exception was the U-Net model, which was trained from random weights according to its standard configuration. The input image size for all models was standardized to 512×512 pixels. To ensure fair comparisons, all models were trained and evaluated under the same experimental environment and a unified data processing pipeline. To validate the stability of our proposed method, the STAR-Net model was trained and evaluated independently three times on all datasets using different random seeds. The performance is reported as the mean and standard deviation (mean ± std) of these runs. For the baseline methods, we followed the common practice in the field and report their performance from a single run. Statistical significance between STAR-Net and the respective second-best performing model (SegFormer MiT-B2 for PlantSeg, DeepLabV3+ResNet-101 for NLB) was assessed using paired t-tests on the primary mIoU metric, based on results from the three independent runs. As significance testing was limited to these two pre-specified comparisons focusing on the closest competitors, no correction for multiple comparisons was applied to the reported p-values. Detailed results, including exact p-values and 95% confidence intervals, are presented in the respective subsection analyses.

### 3.3 Baseline methods

To evaluate our proposed STAR-Net and DPW-Loss, we selected several representative classic and state-of-the-art models as baselines. These methods cover different network architecture paradigms.

First, we selected classic models based on Convolutional Neural Networks (CNNs). These include FCN (Fully Convolutional Networks) (Long et al., 2015), a pioneering work in semantic segmentation (using ResNet-101 (He et al., 2016) as the backbone), and U-Net (Ronneberger et al., 2015), which was designed for biomedical image segmentation and is widely used for its classic encoder-decoder structure and skip connections (using the s5-d16 standard configuration).

Second, we included mainstream models based on atrous convolution and spatial pyramid pooling. This group includes DeepLabV3 (Chen et al., 2017), which uses the ASPP module to capture multi-scale context, and DeepLabV3+ (Chen et al., 2018), which improves upon it with an efficient decoder module for better boundary segmentation. We tested both models with ResNet-50 and ResNet-101 (He et al., 2016) backbones. DeepLabV3+ is a very strong baseline in the field.

Additionally, we included a modern Transformer-based model, SegFormer (Xie et al., 2021). It is an efficient segmentation model that uses a lightweight Mix Transformer (MiT-B2) as its encoder and features a simple all-MLP decoder.

To ensure fairness, all baseline models were trained using their standard or most common loss functions (typically Cross-Entropy loss). Our proposed STAR-Net was trained with DPW-Loss and compared with other loss functions in the ablation study.

### 3.4 Experimental results

This section presents and analyzes the quantitative experimental results of the STAR-Net model and DPW-Loss on the three selected datasets, supplemented by qualitative visualizations for intuitive comparison.

To provide a clear, high-level overview and improve the readability of results spread across multiple tables, we first present two consolidated summary tables.

**Table 3** summarizes the main performance comparison of STAR-Net against all state-of-the-art baselines across the three datasets. Following this, **Table 4** provides a consolidated summary of the key ablation studies, validating our model’s core components.

**Table 3:** Performance comparison of different segmentation methods on the ADLD, PlantSeg, and NLB datasets.

Method	Backbone	ADLD dataset		PlantSeg dataset		NLB dataset	
		mIoU (%)	mAcc (%)	mIoU (%)	mAcc (%)	mIoU (%)	mAcc (%)
U-Net	U-Net (s5-d16)	73.14	74.20	3.14	3.82	77.43	83.63
FCN	ResNet-101	94.29	95.99	9.63	13.28	88.51	92.63
DeepLabV3	ResNet-50	94.05	95.70	17.24	37.95	88.45	93.06
DeepLabV3	ResNet-101	94.18	95.87	20.72	40.63	89.83	93.44
DeepLabV3+	ResNet-50	94.39	95.96	25.08	40.66	89.53	93.79
DeepLabV3+	ResNet-101	94.33	95.96	27.18	42.29	90.35	94.34
SegFormer	MiT-B2	93.36	95.45	40.66	55.24	89.29	93.85
STAR-Net (Ours)	Swin-T	94.63	96.01	41.13	52.67	93.36	96.87

Best results in each column are highlighted in bold. We report the mean results from multiple runs; standard deviations are omitted for clarity.

**Table 4:** Ablation studies on the effectiveness of STAR-Net components (loss function and head architecture).

Study Component	Setting	Dataset	mIoU (%)	mAcc (%)
Loss Function	Cross-Entropy (CE)	PlantSeg	35.74	46.62
	Generalized Dice (GDL)		34.10	45.27
	Focal Loss (FL)		37.48	49.72
	Combo Loss		39.34	51.95
	DPW-Loss (Ours)		**41.13 ± 0.13**	**52.67 ± 0.13**
Head Architecture	ASPP + STAR-Net Decoder	NLB	91.25	95.18
	HBAA + STAR-Net Decoder (Ours)		**93.36**	**96.87**

Best results for each study are in bold.

The detailed breakdown of results for each specific dataset and further in-depth analyses are then presented in the subsequent subsections.

#### 3.4.1 Performance on the ADLD dataset

We first evaluated model performance on our self-made ADLD dataset. This dataset is intended to provide a clear benchmark scenario for plant disease segmentation. As shown in **Table 5**, our proposed STAR-Net (Swin-T, with *L_CE_*) performed exceptionally well on this benchmark, achieving an average mIoU of 94.63% (± 0.07) and an average mAcc of 96.01% (± 0.05), the most competitive results among all compared methods. We also observed that all advanced models achieved high scores on this dataset (mIoU generally above 93%). This indicates that the ADLD dataset is an ideal scenario for validating and comparing the fundamental segmentation capabilities of various models. The leading performance of STAR-Net on this dataset demonstrates the effectiveness of its architecture and lays a solid foundation for its evaluation on more complex datasets.

**Table 5:** Performance comparison of different segmentation methods on the ADLD dataset.

Method	Backbone	mIoU (%)	mAcc (%)
U-Net	U-Net (s5-d16)	73.14	74.20
FCN	ResNet-101	94.29	95.99
DeepLabV3	ResNet-50	94.05	95.70
DeepLabV3	ResNet-101	94.18	95.87
DeepLabV3+	ResNet-50	94.39	95.96
DeepLabV3+	ResNet-101	94.33	95.96
SegFormer	MiT-B2	93.36	95.45
STAR-Net (Ours)	Swin-T	94.63 ± 0.05	96.01 ± 0.03

#### 3.4.2 Performance on the PlantSeg dataset

The PlantSeg dataset poses a severe test for segmentation models due to the complexity of its “in-the-wild” images, the large number of disease classes (115), and the diverse forms of disease expression. The results in **Table 6** show that traditional models (U-Net, FCN) performed poorly on this highly challenging dataset. While models like DeepLabV3+ showed improvement, their performance was still limited. The Transformer-based SegFormer achieved an mIoU of 40.66%, demonstrating the competitiveness of modern architectures. Our proposed STAR-Net (with Swin-T backbone and trained with DPW-Loss) achieved an average mIoU of 41.13% and an average mAcc of 52.67%. Its performance is comparable to SegFormer and significantly better than the DeepLab series. Given the extreme complexity of this dataset, this result strongly validates the combined effectiveness of the STAR-Net architecture and the DPW-Loss training strategy. Furthermore, a paired t-test confirmed that the improvement in mIoU achieved by STAR-Net over SegFormer (MiT-B2), the second-best performing model on this dataset, is statistically significant (p = 0.0002). The 95% confidence interval for the mean difference was [0.45, 0.49] percentage points, indicating a highly consistent, albeit modest, advantage for STAR-Net on this challenging dataset.

**Table 6:** Performance comparison of different segmentation methods on the PlantSeg dataset.

Method	Backbone	mIoU (%)	mAcc (%)
U-Net	U-Net (s5-d16)	3.14	3.82
FCN	ResNet-101	9.63	13.28
DeepLabV3	ResNet-50	17.24	37.95
DeepLabV3	ResNet-101	20.72	40.63
DeepLabV3+	ResNet-50	25.08	40.66
DeepLabV3+	ResNet-101	27.18 ± 0.15	42.29 ± 0.18
SegFormer	MiT-B2	40.66 ± 0.14	55.24 ± 0.11
STAR-Net (Ours)	Swin-T	41.13 ± 0.13	52.67± 0.13

#### 3.4.3 Performance on the NLB dataset

The NLB dataset focuses on Northern Leaf Blight in corn, featuring a single disease class with relatively well-defined characteristics. As shown in **Table 7**, the performance of all modern models improved significantly on this dataset. Our proposed STAR-Net (Swin-T, with *L_CE_*) was particularly outstanding, achieving an average mIoU of 93.36% and an mAcc of 96.87%, the highest mIoU among all compared models. This leading performance indicates our model has a significant advantage in handling specific morphological features, such as the elongated lesions in the NLB dataset. Furthermore, a paired t-test confirmed that the improvement in mIoU achieved by STAR-Net over DeepLabV3+ (ResNet-101), the second-best performing model on this dataset, is statistically significant (p = 0.0012). The 95% confidence interval for the mean difference was [2.56, 3.46] percentage points, highlighting a substantial and statistically robust performance gain.

**Table 7:** Performance comparison of different segmentation methods on the NLB dataset.

Method	Backbone	mIoU (%)	mAcc (%)
U-Net	U-Net (s5-d16)	77.43	83.63
FCN	ResNet-101	88.51	92.63
DeepLabV3	ResNet-50	88.45	93.06
DeepLabV3	ResNet-101	89.83	93.44
DeepLabV3+	ResNet-50	89.53	93.79
DeepLabV3+	ResNet-101	90.35 ± 0.10	94.34 ± 0.09
SegFormer	MiT-B2	89.29 ± 0.12	93.85 ± 0.07
STAR-Net (Ours)	Swin-T	93.36 ± 0.08	96.87 ± 0.06

#### 3.4.4 Effectiveness of DPW-Loss (ablation study)

To independently validate the effectiveness of DPW-Loss, we conducted an ablation study on the most challenging PlantSeg dataset using the STAR-Net architecture. We compared our method with several common single-component loss functions (e.g., CE, GDL, Focal Loss) as well as a representative hybrid framework, ComboLoss (TaGhanaki et al., 2019). As shown in **Table 8**, when trained with DPW-Loss, the model achieved an average mIoU of 41.13%, significantly outperforming all baselines. Notably, DPW-Loss surpasses not only the best-performing single-component loss, Focal Loss (*L_Focal_*, mIoU 37.48%), but also the strong hybrid baseline, ComboLoss (mIoU 39.34%). Compared to the next-best-performing ComboLoss, DPW-Loss provided a notable 1.79 percentage point increase in mIoU. This result strongly demonstrates the superiority of the DPW-Loss design. Its dynamic and synergistic optimization mechanism enables the model to more comprehensively address the multiple challenges in complex segmentation tasks, thereby achieving better performance than both standard and advanced hybrid losses.

**Table 8:** Performance comparison of different loss functions with the STAR-Net model on the PlantSeg dataset.

Loss Function	mIoU (%)	mAcc (%)
Cross-Entropy Loss (CE)	35.74	46.62
Generalized Dice Loss (GDL)	34.10	45.27
Focal Loss (FL)	37.48	49.72
Combo Loss	39.34	51.95
DPW-Loss (Ours)	41.13 ± 0.13	52.67± 0.13

#### 3.4.5 Effectiveness of the head architecture (ablation study)

To isolate and rigorously validate the unique contribution of our proposed Heterogeneous Branch Attention Aggregation (HBAA) module, we conducted a critical ablation study. We replaced our HBAA module with a strong baseline, the ASPP module. All other components, including the Swin-T backbone, decoder, and DPW-Loss strategy, remained identical.

We chose the NLB dataset for this experiment because its core challenge is segmenting dense, elongated lesions. This is precisely the problem our HBAA module’s Strip Pooling branch was designed to solve. This targeted comparison can most clearly demonstrate the effectiveness of our specialized design philosophy over general-purpose multi-scale approaches.

The results, presented in **Table 9**, show a significant performance gain for our design. The model equipped with our HBAA module achieved an mIoU of 93.36%, surpassing the powerful ASPP-equipped baseline (91.25%) by a margin of 2.11 percentage points. This result provides strong quantitative evidence. It shows that for specific lesion morphologies in agriculture, our heterogeneous feature aggregation approach is more advantageous. For this challenging task, our approach proved superior to a monolithic, general-purpose fusion module. This success is due to the synergy between the Strip Pooling branch, which preserves long lesion integrity, and the Window Attention branch, which captures fine-grained local textures.

**Table 9:** Ablation study on the effectiveness of the proposed head architecture on the NLB dataset.

Head architecture	mIoU (%)	mAcc (%)
ASPP + STAR-Net Decoder	91.25	95.18
HBAA + STAR-Net Decoder (Ours)	93.36	96.87

To further validate the effectiveness of our heterogeneous design and the synergy mentioned above, we conduct a second ablation study on the key components within the HBAA module. Specifically, we remove the Window Attention branch and the Strip Pooling branch individually to observe their impact on performance.

The results are presented in **Table 10**. Our full HBAA model (Baseline) achieves the best performance with 93.36% mIoU and 96.87% mAcc. When the Window Attention branch is removed, the mIoU drops by 0.78% to 92.58%. Similarly, removing the Strip Pooling branch—which is critical for elongated lesions—leads to a more significant performance degradation, with mIoU decreasing by 1.97% to 91.39%. This experiment confirms that both components are essential and contribute positively to the module’s effectiveness, justifying the necessity of our heterogeneous design.

**Table 10:** Ablation study on the components of the HBAA module.

Configuration	mIoU (%)	Δ (%)
HBAA (Full Model)	93.36	–
w/o Window Attention	92.58	-0.78
w/o Strip Pooling	91.39	-1.97

The ▵ column shows the performance change compared to the full model.

#### 3.4.6 Model interpretability analysis

**Figure 6:**
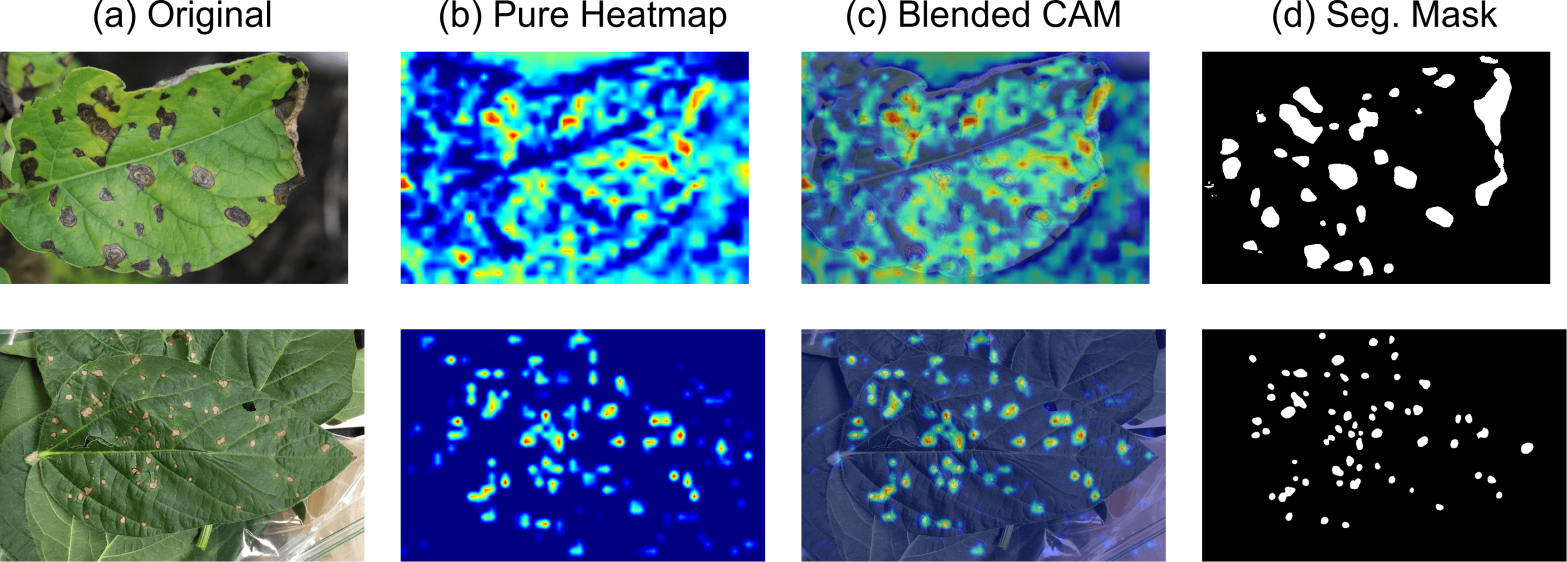
Grad-CAM visualizations for STAR-Net. This figure illustrates STAR-Net’s interpretability and ability to focus on disease-affected regions. **(a)** Original input image. **(b)** Pure heatmap generated by Grad-CAM, highlighting regions critical for the classification decision. **(c)** Blended CAM, which overlays the heatmap onto the original image, providing clear visual context. **(d)** Ground-truth segmentation mask, serving as a reference for the actual disease locations.

To validate that our model’s high performance is based on relevant evidence and not spurious correlations, we analyzed its internal feature responses using Grad-CAM. The visualizations, presented in **Figure 6**, provide critical insights into the model’s decision-making process.

A detailed inspection of the ‘Pure Heatmap’ (b) and ‘Blended CAM’ (c) reveals that the model’s high-activation regions (indicated by warm colors) are precisely concentrated on the actual disease spots on the leaves. This observed focus aligns remarkably well with the ground-truth segmentation masks (d) and contrasts sharply with the background, which remains unactivated (cool colors).

#### 3.4.7 Qualitative analysis (visual comparison)

**Figure 7:**
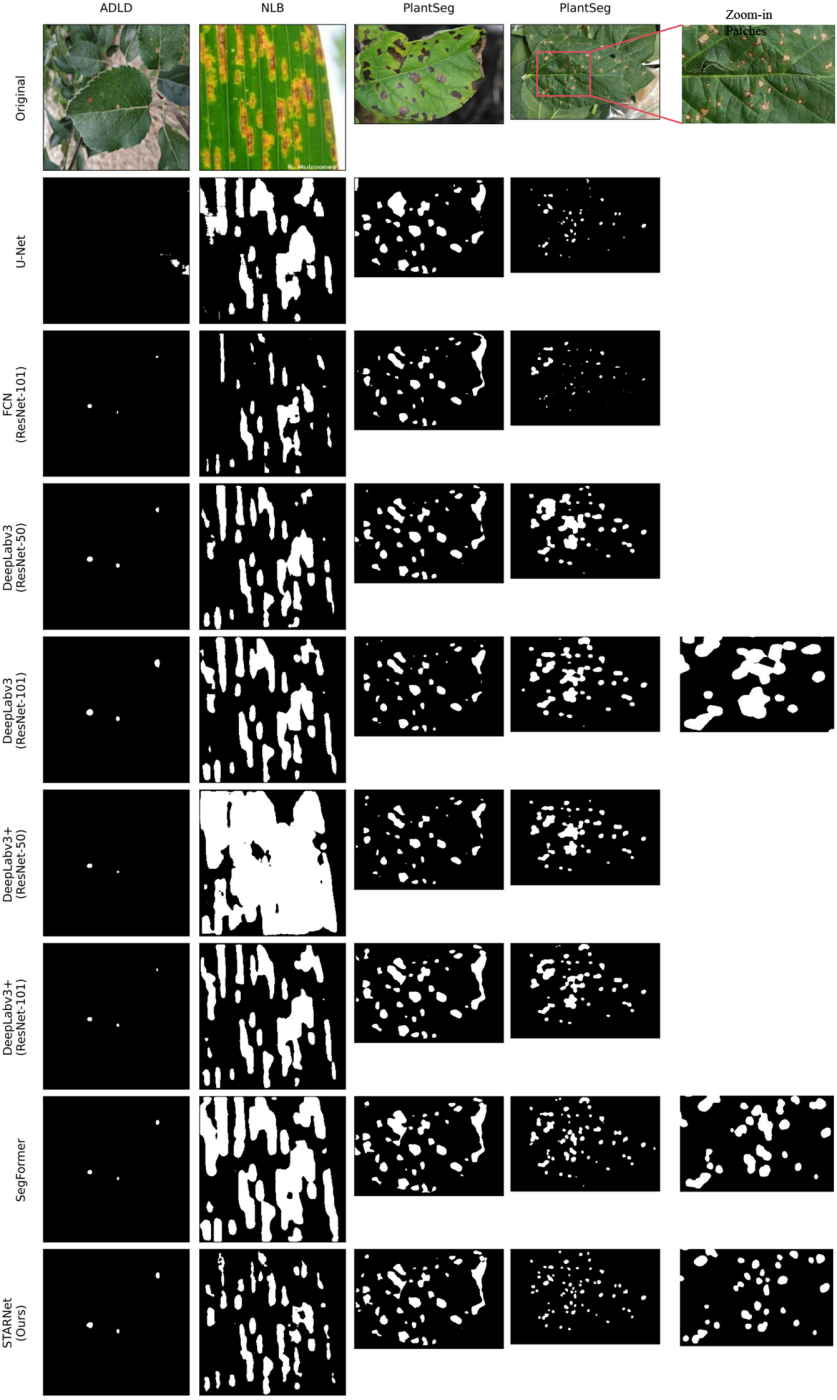
Visual comparison of segmentation results from different models on the ADLD, NLB, and PlantSeg datasets. The first row shows the original images, and subsequent rows display the segmentation masks from the respective models.

To more intuitively assess model performance, we visualized the segmentation results of different models on the three datasets (ADLD, NLB, PlantSeg), as shown in **Figure 7**. The figure juxtaposes the original images with the segmentation masks from STAR-Net and several mainstream baseline models. The visualizations provide strong evidence of the significant superiority and robustness of our proposed STAR-Net in handling a variety of complex plant disease images.

The specific observations and analysis are as follows:

- On the ADLD dataset: In the relatively simple scenarios represented by our self-developed dataset, some earlier or lightweight models (e.g., U-Net, FCN, DeepLabV3) performed poorly, with severe missed detections or misidentifications. Strong baselines like DeepLabV3+ (ResNet 101) and SegFormer also achieved excellent segmentation. However, STAR-Net reached the same top-tier level, segmenting lesions precisely and demonstrating robust performance.
- On the NLB dataset: The challenge here is to accurately segment dense and elongated, strip-like lesions. A common problem with baseline models is “lesion adhesion.” This occurs when multiple independent lesions are incorrectly merged into a single area. This phenomenon was particularly evident in the DeepLab series and SegFormer. The core advantage of our model is that STAR-Net avoids adhesion while preserving the structural integrity of each lesion. This success is mainly due to two components of the HBAA module. The strip pooling branch strongly models elongated shapes, while the window attention branch depicts fine-grained detail, resulting in a more refined segmentation.
- On the PlantSeg dataset: This dataset represents the most challenging scenario, with minute and highly dense lesions. As seen in the two samples, most baseline models could identify the majority of lesions but suffered from severe adhesion, failing to clearly separate individual small fragments. In contrast, STAR-Net’s core advantage is again evident. It detects the vast majority of tiny lesions and successfully avoids adhesion, maintaining their structural integrity. This again proves that the multi-branch design of the HBAA module effectively aggregates multi-scale information. DPW-Loss also played a key role. Its emphasis on boundary optimization in later training stages led to the most refined segmentation with the least background noise.

In summary, the visual comparison shows STAR-Net’s superior performance. The model excels at both precise segmentation in regular scenarios and robust identification in challenging scenes.

**Figure 8:**
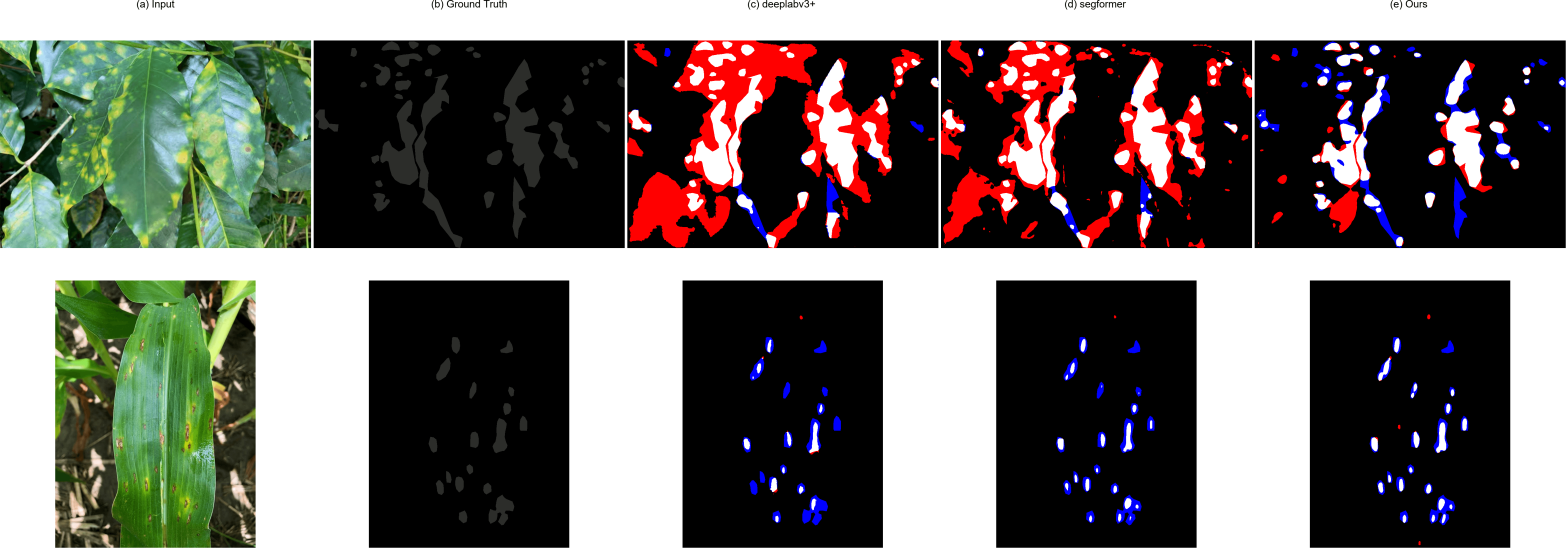
Quantitative visualization of segmentation results using IoU maps. **(a)** Input Image. **(b)** Ground Truth. **(c)** DeepLabV3+. **(d)** SegFormer. **(e)** Ours. For the maps **(c–e)**, white pixels indicate True Positives (TP), red pixels indicate False Negatives (FN), and blue pixels indicate False Positives (FP). Our method **(e)** demonstrates fewer FP and FN regions, indicating a more accurate boundary adherence.

To provide a more detailed, pixel-level analysis of segmentation performance, we visualize the segmentation errors using IoU maps in **Figure 8**. These maps illustrate the pixel-level agreement between the model predictions and the ground truth. As defined in the caption, white, red, and blue pixels represent True Positives (TP), False Negatives (FN), and False Positives (FP), respectively. As shown, the baseline models such as DeepLabV3+ and SegFormer exhibit noticeable FN (red) and FP (blue) regions, indicating missed lesion areas or over-segmentation. In contrast, our STAR Net (e) produces significantly cleaner maps with minimal FP/FN errors, which visually confirms its superior accuracy in boundary adherence.

### 3.5 Computational efficiency analysis

In addition to segmentation accuracy, computational efficiency is a critical factor for the practical application of deep learning models in precision agriculture, especially for deployment on edge devices. To provide a comprehensive and fair evaluation of STAR-Net’s practicality, we compared its computational complexity and key efficiency metrics against the main baseline models. To ensure a fair comparison, we explicitly note that all efficiency metrics (FLOPs, Latency, etc.) were benchmarked under a unified standard: a 512 × 512 input resolution and a batch size of 1. The detailed results are presented in **Table 11**.

**Table 11:** Comparison of computational efficiency.

Method	Backbone	Params (M)	FLOPs (G)	mIoU (PlantSeg) %	Memory (MB)	Latency (ms)
DeepLabV3+	ResNet-101	58.6	83.4	27.18	3584	21.1
SegFormer	MiT-B2	27.5	50.6	40.66	2253	17.8
SegFormer	MiT-B3	47.3	84.7	41.95	4158	22.6
STAR-Net (Ours)	Swin-T	32.5	30.0	41.13	3126	19.7

All metrics are calculated for a 512 × 512 input with a batch size of 1 on a single NVIDIA RTX 4090 GPU. The mIoU on the challenging PlantSeg dataset is included to evaluate the performance-efficiency trade-off.

The results highlight that our proposed STAR-Net demonstrates a remarkable balance between high performance and computational efficiency. Compared to DeepLabV3+ (ResNet-101), STAR-Net is more efficient. It requires less than half the computational resources and fewer parameters, while achieving higher accuracy on the challenging PlantSeg dataset.

**Figure 9:**
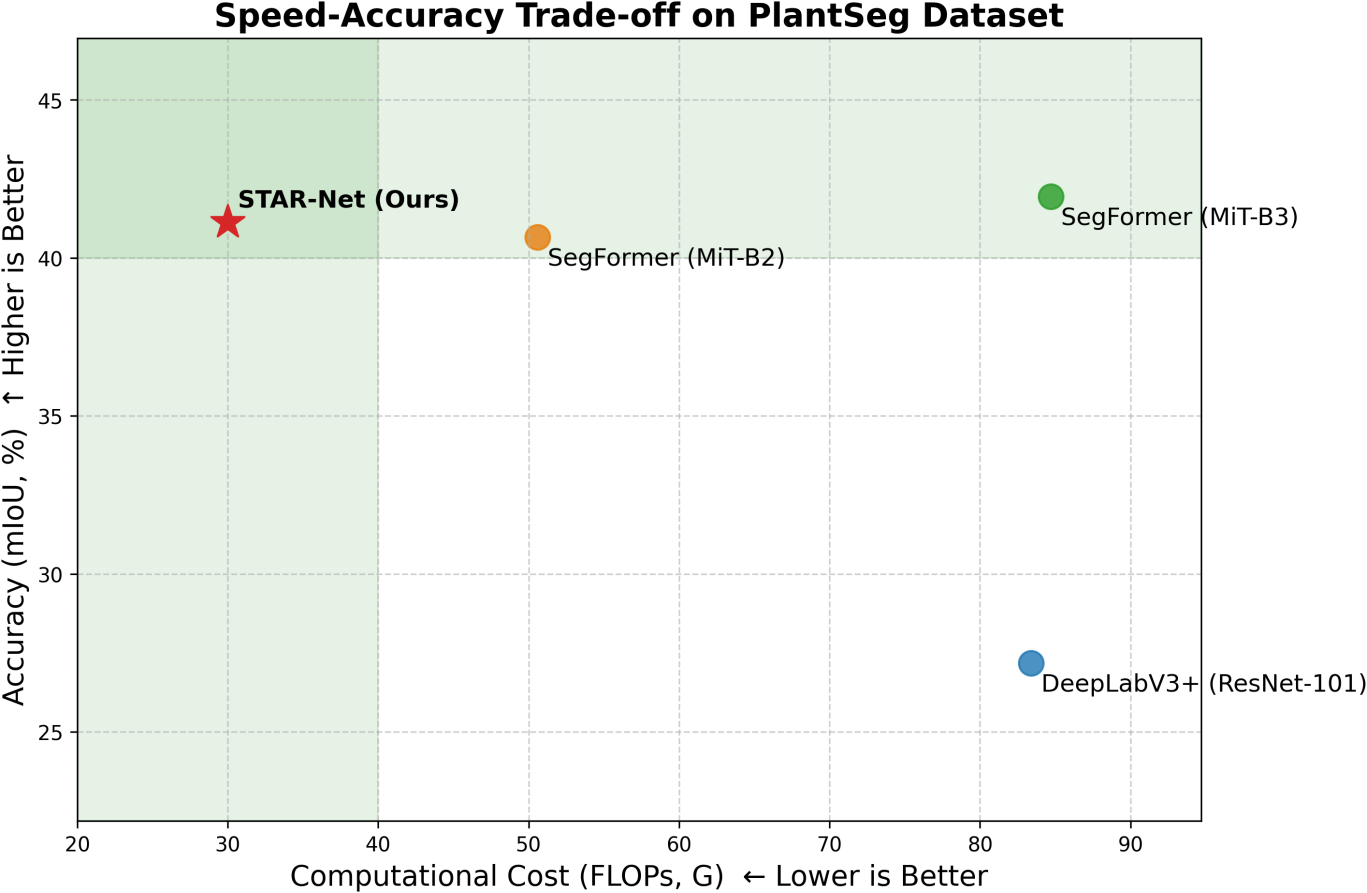
Speed-accuracy trade-off on PlantSeg dataset. Our model (STAR-Net) is positioned in the optimal top-left quadrant, indicating high accuracy (mIoU) with low computational cost (GFLOPs).

More importantly, the comparison with the modern Transformer-based SegFormer series provides a clear accuracy-speed tradeoff analysis. To intuitively demonstrate this tradeoff, we have incorporated the speed-accuracy curve in **Figure 9**. This figure charts the computational cost (GFLOPs) against segmentation accuracy (mIoU), placing the most desirable models in the top-left quadrant (high accuracy, low cost). As can be clearly seen, our STAR-Net (Ours) is positioned firmly in this optimal region, demonstrating a superior balance of performance and efficiency over the other methods.

This visual evidence is supported by the specific metrics: The lighter SegFormer (MiT-B2) offers the fastest inference (17.8 ms) but at the cost of lower accuracy. Our STAR-Net (19.7 ms) achieves a better mIoU (41.13% vs 40.66%) with a considerably lower computational cost (30.0G vs. 50.6G FLOPs). The comparison with the larger SegFormer (MiT-B3) is even more telling. STAR-Net uses only 70% of the parameters and 35% of the computational cost (FLOPs) of MiT-B3. Despite this efficiency, it delivers a highly competitive accuracy (41.13% vs. 41.95%) at a faster speed (19.7 ms vs. 22.6 ms).

This detailed and visually-supported analysis proves our architecture is both accurate and highly efficient. This high efficiency-to-performance ratio makes STAR-Net an ideal candidate for future lightweighting and deployment on resource-constrained edge devices (e.g., Jetson series or mobile SoCs), where balancing performance and resource consumption is paramount.

## 4 Discussion

This section provides a deeper interpretation of the preceding experimental results. We will synthesize the performance of STAR-Net, conduct a critical analysis of its limitations, especially on challenging “in-the-wild” data, and discuss the practical implications and future directions of our work.

### 4.1 Synergistic advantages of architecture and training strategy

Our experimental results show that our integrated approach is effective. Its success stems from the synergy between architectural innovation and a dynamic training strategy.

The primary architectural innovation, the HBAA module, was specifically engineered to capture heterogeneous lesion features. Its targeted design was validated on the NLB dataset, where STAR-Net achieved a state-of-the-art mIoU of 93.36% by substantially mitigating the “lesion adhesion” problem that plagued competing models.

Building on this powerful foundation, the DPW-Loss strategy proved crucial for tackling the chaotic “in-the-wild” scenario of the PlantSeg dataset. The significant 1.79 percentage point mIoU gain from DPW-Loss highlights the importance of its phase-aware synergistic mechanism. The strategy mimics an expert’s learning process. It stabilizes with *L_CE_*, addresses imbalance with *L_Focal_*, and refines boundaries with *L_GDL_*, effectively unlocking the architecture’s potential. This synergy resulted in a competitive mIoU of 41.13% on a highly complex dataset. It showcases that our integrated approach can generalize from specific morphological challenges to diverse, imbalanced environments.

### 4.2 Critical analysis of performance gap and limitations

A critical scientific appraisal requires analyzing the performance disparity between the NLB (93.36% mIoU) and the highly challenging PlantSeg (41.13% mIoU) datasets. This performance gap does not simply reflect the dataset’s difficulty but reveals fundamental limitations of the current paradigm, which we address below.

Root Cause Analysis — Task Complexity and Long-Tail Distribution: The primary reason for the performance drop is an exponential increase in task complexity. The NLB dataset represents a near-binary segmentation task for a single disease, whereas PlantSeg is an extremely challenging 115-class fine-grained segmentation task. Our analysis confirms the PlantSeg dataset has a long-tail distribution. The pixel count for the most common diseases is thousands of times greater than for the rarest classes. The mIoU metric weights each class equally. Therefore, poor performance on data-sparse classes, where IoU scores can be near zero, significantly depresses the average score.

**Figure 10:**
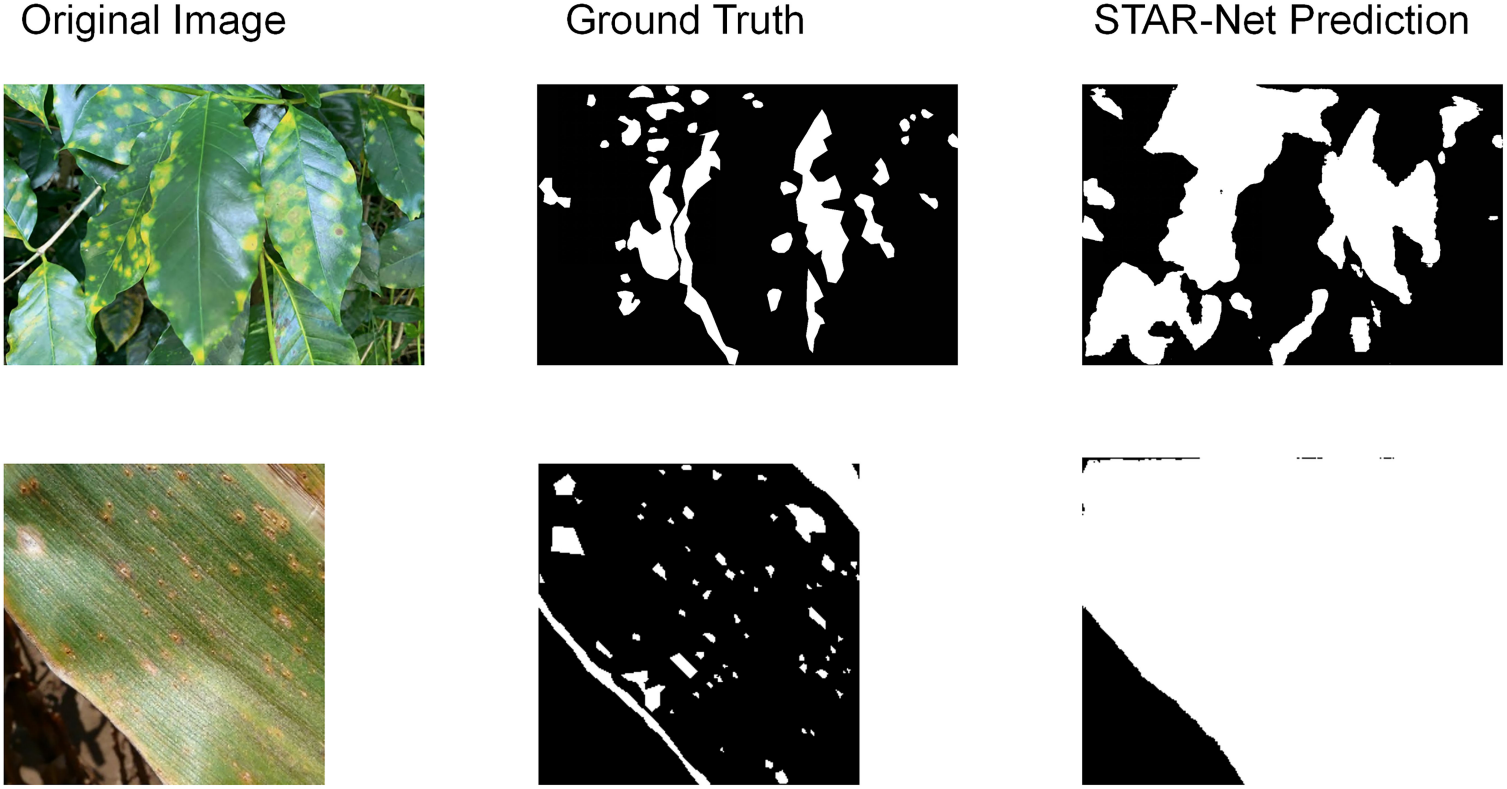
Typical failure cases of STAR-Net.

Analysis of Specific Failure Cases: A qualitative analysis of our model’s characteristic failure modes on the PlantSeg dataset reveals two primary challenges, illustrated in **Figure 10**. The first and most significant issue stems from the dataset’s long-tail distribution: failure to segment rare diseases. The model struggles with classes in the long tail that have few training samples. It fails to learn effective features, often resulting in false positives where healthy regions are misclassified as lesions. The second common issue is the adhesion of small and dense lesions. STAR-Net improves upon baselines, but it still cannot perfectly separate extremely small, dense lesions. It often merges them into a single, larger region.

To systematically supplement and quantify these qualitative observations, we conducted a quantitative failure analysis. We defined a “failure case” as any image in our 2,294-image test set where the prediction achieved an IoU score below the standard 0.5 threshold. This criterion revealed that 1,331 images (approx. 58%) fall into this category, quantitatively confirming the dataset’s extreme difficulty.

**Figure 11:**
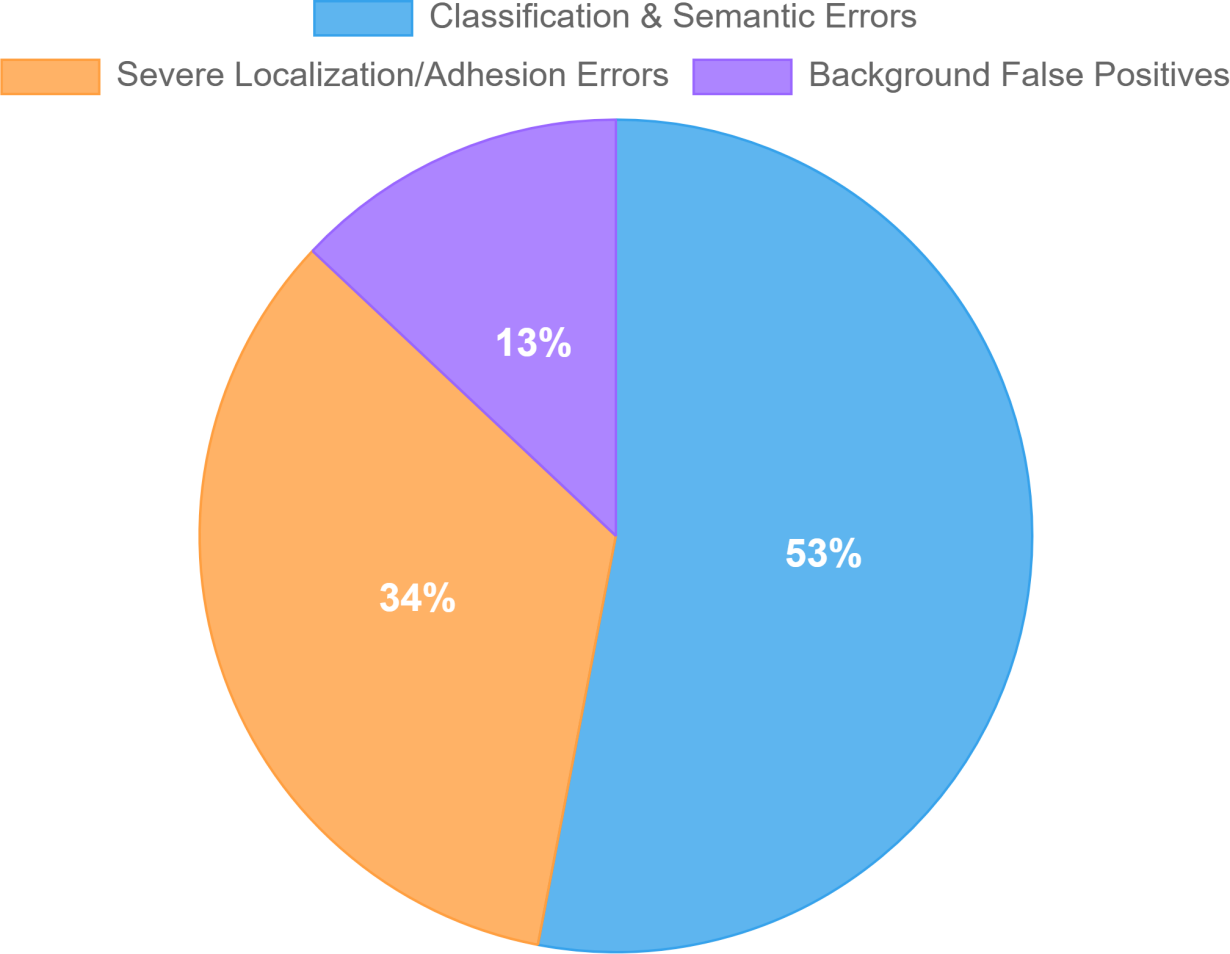
A quantitative breakdown of dominant failure modes on the PlantSeg test set, based on a random sample of 100 images with IoU < 0.5. This chart complements the qualitative examples also shown.

From this large failure pool, we randomly sampled 100 images for manual categorization, assigning each to its single, most dominant error. The resulting distribution (visualized in the chart in **Figure 11**) identifies three primary failure modes, which align closely with our qualitative findings:

Classification & Semantic Errors (53%): This was the most dominant failure mode. It quantitatively confirms the “failure to segment rare diseases” challenge, including cases where the model fundamentally misidentified a class, confused two similar classes, or missed a rare class entirely.

Severe Localization/Adhesion Errors (34%): This category directly corresponds to the “adhesion” challenge. It refers not to minor boundary inaccuracies, but to severe localization failures where the model so completely merged distinct disease instances that the mask’s IoU dropped below 0.5.

Background False Positives (13%): This category includes cases where non-leaf areas (e.g., soil, background clutter) were incorrectly identified as disease.

Reflection on the Limitations of the Current Paradigm: This significant performance gap leads to a crucial reflection: architectural innovation alone may be insufficient to bridge this gap. STAR-Net shows progressive improvements. However, achieving true robustness in unconstrained agricultural environments may require a fundamental shift beyond network design. This honest appraisal suggests that future breakthroughs will likely depend on advances in data representation, pre-training strategies, and learning paradigms.

### 4.3 Practical applications and future work

To bridge the gap between our technical achievements and real-world impact, we propose a concrete application pipeline and outline key directions for future research.

A Concrete Application Pipeline: Our STAR-Net model can be integrated into a drone-based automated field scouting system. The workflow would involve: (1) Large-scale image acquisition by drones; (2) Real-time analysis on an edge-computing device using a lightweight version of STAR-Net; and (3) Generation of a “disease heatmap” of the field, which can be visualized in farm management software to guide growers toward targeted interventions.

An Actionable Metric — Disease Severity Index (DSI): To make the model’s output directly useful for agronomists, the pixel-level segmentation can be translated into a quantitative metric. By calculating the ratio of lesion pixels to total leaf pixels within a region of interest (DSI = (Lesion Area/Leaf Area) * 100%), we can derive a DSI. This index can set alert thresholds (e.g., DSI > 5%) to automatically trigger precision spraying. This directly links our technology to the ‘diagnose-decide-act’ loop in precision agriculture.

Future Research Directions: Based on our findings, we have identified several key areas for future work.

- Model Lightweighting: A crucial next step is to investigate model compression techniques like knowledge distillation or pruning. The goal is to develop a lightweight version of STAR-Net for resource-constrained edge devices.
- Advanced Learning Paradigms: To address the long-tail data problem, exploring new paradigms such as data-efficient training (e.g., self-supervised and semi-supervised learning), few-shot, zero-shot, or open-set learning is essential for improving performance on rare diseases.
- Large-Scale Pre-training and Multi-modal Fusion: Developing a foundational model for agriculture through large-scale, domain-specific pre-training could significantly boost robustness. Furthermore, fusing our visual-based model (RGB) with other data modalities, such as hyperspectral or thermal infrared imagery, could provide richer diagnostic information.

## 5 Conclusion

In this paper, we presented an integrated solution to the multifaceted challenge of plant disease segmentation in complex, real-world agricultural environments. Our work is founded on a key principle: robust performance requires a synergistic combination of architectural innovation and an intelligent training strategy. We introduced STAR-Net, a novel architecture with a Heterogeneous Branch Attention Aggregation (HBAA) module. It proved highly effective at capturing diverse lesion morphologies and mitigating common segmentation errors. To optimize its training, we proposed the Dynamic Phase-Weighted Loss (DPW-Loss), a strategy that adaptively navigates the challenges of extreme class imbalance. Our extensive experiments validate our integrated approach. It achieves highly competitive performance across diverse datasets and is a powerful, robust solution with significant potential for precision agriculture.

## Data Availability

The dataset presented in this study was compiled from images aggregated from various publicly available web resources, including open-source platforms (e.g., Kaggle). Due to the diverse and often unspecified copyright terms of the original source images, the authors do not have the permission to redistribute the compiled dataset. Requests to access the datasets should be directed to YF, a2858198772@163.com.
